# Dysregulated Recruitment of the Histone Methyltransferase EZH2 to the Class II Transactivator (CIITA) Promoter IV in Breast Cancer Cells

**DOI:** 10.1371/journal.pone.0036013

**Published:** 2012-04-26

**Authors:** Agnieszka D. Truax, Meghna Thakkar, Susanna F. Greer

**Affiliations:** Division of Cellular and Molecular Biology and Physiology, Department of Biology, Georgia State University, Atlanta, Georgia, United States of America; University of Medicine and Dentistry of New Jersey, United States of America

## Abstract

One mechanism frequently utilized by tumor cells to escape immune system recognition and elimination is suppression of cell surface expression of Major Histocompatibility Class II (MHC II) molecules. Expression of MHC II is regulated primarily at the level of transcription by the Class II Transactivator, CIITA, and decreased CIITA expression is observed in multiple tumor types. We investigate here contributions of epigenetic modifications to transcriptional silencing of CIITA in variants of the human breast cancer cell line MDA MB 435. Significant increases in histone H3 lysine 27 trimethylation upon IFN-γ stimulation correlate with reductions in transcription factor recruitment to the interferon-γ inducible CIITA promoter, CIITApIV, and with significantly increased CIITApIV occupancy by the histone methyltransferase enhancer of zeste homolog 2 (EZH2). Most compelling is evidence that decreased expression of EZH2 in MDA MB 435 variants results in significant increases in CIITA and *HLA-DRA* mRNA expression, even in the absence of interferon-γ stimulation, as well as increased cell surface expression of MHC II. Together, these data add mechanistic insight to prior observations of increased EZH2 expression and decreased CIITA expression in multiple tumor types.

## Introduction

Major histocompatibility class II (MHC II) genes encode cell surface proteins responsible for presenting extracellularly derived peptides for activation of CD4^+^ T cells. As activated T cells are in turn responsible for driving adaptive immune responses, MHC II molecules play critical roles in regulating immune recognition of pathogens and tumors. Constitutive expression of MHC class II is limited to professional antigen presenting cells, but MHC II expression is induced in all nucleated cells by inflammatory cytokines, of which IFN-γ is the most potent [1], [2]. Although CD8^+^ T cells are directly responsible for lysis of infected cells and tumor cells, recent studies have shown that peptide immunization in the presence of CD4^+^ T cells enhances CD8^+^ T cell responses [3]. Further, several murine tumor models have demonstrated that CD4^+^ T cells are required for an effective anti-tumor immune response [4], [5], [6], [7]. Loss of MHC II expression is associated with decreased numbers of tumor infiltrating T cells and with increased tumor aggressiveness [8], [9]. Together, these observations suggest CD4^+^ T cells respond to tumor antigens presented via MHC II to induce an effective immune response and emphasize the importance of transcriptional regulation of MHC II genes in cancer cells.

Tight regulation of MHC II transcription is necessary for proper initiation, stabilization, and termination of adaptive immune responses to infection and to tumors. MHC II genes are regulated by a multi-protein enhanceosome complex that binds the W-X-Y region of *HLA-DRA* promoters, assembly of which is stabilized by the Class II transactivator, CIITA [10], [11]. While CIITA does not directly bind MHC II promoters, its association with the pre-assembled enhanceosome complex is required for MHC II expression and serves to coordinate steps leading to transcriptional initiation [12], [13]. CIITA recruits to MHC II promoters, including the *HLA-DRA* proximal promoter utilized in this study, components of the basal transcriptional machinery, histone acetyltransferases (HATs), histone deacetylases (HDACs), chromatin remodeling complexes, and kinases that phosphorylate RNA pol II [14], [15], [16], [17], [18], [19].

CIITA transcription is also tightly regulated in a cell specific manner from four distinct promoters [20], [21]. Promoter I drives expression of CIITA in dendritic cells [22], the function of promoter II is unknown, and promoter III drives constitutive CIITA expression in B cells but can also be up regulated with cytokine stimulation [23], [24], [25]. Transcription of CIITA in non-antigen presenting cells is induced by IFN-γ by orchestrated binding of multiple transcription factors to the promoter IV isoform of CIITA (CIITApIV) [24]. Transcriptional activation of CIITApIV by IFN-γ requires the assembly of interferon regulatory factor 1 (IRF-1), signal transducer and activator of transcription 1 (STAT-1), and ubiquitous factor 1 (USF-1) [26]. STAT-1 directly binds ubiquitously expressed USF-1 at the E-box of the IFN-γ activated sequence (GAS). STAT-1 also initiates transcription from the IRF-1 promoter; once IRF-1 is expressed, it subsequently binds the IFN response element (IRE) site at CIITApIV [25].

Previous studies from our lab and others indicate that epigenetic modifications to chromatin play important roles in regulating transcription of *HLA-DRA* and CIITApIV genes [27], [28], [29]. In unstimulated cells, the *HLA-DRA* promoter exhibits low basal acetylation which allows for binding of the ubiquitously expressed components of the enhanceosome complex. Following cytokine stimulation, acetylation of histones (H) H3 and H4 significantly increases, allowing recruitment of CIITA and the basal transcription machinery and initiation of MHC II transcription [15], [30]. CIITApIV is also regulated by multiple epigenetic modifications and is characterized as a bivalent promoter with both activating and repressing chromatin marks. In unstimulated cells, CIITApIV exhibits elevated trimethylation of histone H3 lysine 27 (H3K27me3) and low acetylation of histones H3 and H4. In the presence of IFN-γ, changes in higher order chromatin structure are followed by increases in acetylation of histones H3 and H4 [31], [32], increased trimethylation of histone H3 lysine 4 (H3K4me3) [33], and a significant and rapid decrease in H3K27me3 [32], [34], [35]. The histone methyltransferase (HMTase) largely responsible for the addition of methyl groups to H3K27 is the Enhancer of Zeste Homolog 2 (EZH2) [36], [37], the catalytic subunit of the Polycomb repressive complex 2 (PRC2) which is involved in maintaining epigenetic memory and transcriptional silencing [36], [38]. While EZH2 has previously been shown to bind CIITApIV in malignant uveal melanoma cells, mechanistic roles for EZH2 in regulating CIITA and MHC II expression remain to be elucidated. Indeed, although studies have demonstrated that EZH2 is frequently overexpressed in a wide variety of cancers including tumors of the prostate and breast, mechanistic links of EZH2 to cancer progression remain areas of intense investigation.

Decreased expression of MHC II has been previously described in breast tumors [9], [39]. Variants of the MDA MB 435 human breast cancer cell line differ in their metastatic ability [40] and have recently been classified as members of the HER-2 overexpressing subtype of breast carcinoma [41]. Previous work has demonstrated that among these variant lines, 435 Brain 1 cells exhibit poor metastatic ability while 435 Lung 2 cells exhibit high metastatic ability [40], [42]. We provide additional evidence for epigenetic regulation of CIITA linked to the decreased expression of cell surface MHC II in highly metastatic breast cancer cells. In this study, we analyzed MHC II and CIITA expression patterns in variants of MDA MB 435. Initial studies determined a loss of cell surface MHC II in the MDA MB 435 variants which correlated with increased metastatic potential, decreased CIITA expression, and suppression of CIITApIV. We provide evidence that in the MDA MB 435 variants, CIITApIV maintains a closed chromatin conformation in the presence or absence of IFN-γ stimulation. Elevated levels of EZH2 at CIITApIV and the resulting increases in CIITApIV H3K27me3 occur in the presence of IFN-γ and leave the proximal promoter inaccessible for transcription factor binding or transcription initiation. Decreased expression of EZH2 results in expression of CIITA and MHC II, even in unstimulated cells, and has substantial impact on MHC II cell surface expression in each of the MDA MB 435 variants. These observations suggest EZH2 is the pivotal regulator of CIITApIV silencing which is in agreement with our previous observations [29] and implicate EZH2 as a candidate target for use in activating immune responses. We further provide novel evidence that EZH2 contributes critical regulatory marks specifically to the silencing of CIITA transcription and to the regulation of MHC II expression in MDA MB 435 breast cancer cell variants, indicating EZH2 may play a role in promoting tumor growth through immunoevasion in the presence of inflammatory cytokine.

## Materials and Methods

### Cells

435-Lung 2 and 435-Brain 1 variants of the estrogen independent MDA MB 435 human breast cancer cell line were provided by Dr. Janet Price [42]. These cell lines were sent without identifiers with a Materials Transfer Agreement (MTA) (M.D. Anderson #6077) governing the transfer and use of these cell lines. Per institutional policy, M.D. Anderson MTA#6077 was reviewed and approved by the M.D. Anderson IRB on March 23, 2009. The IRB determined that the use of these cell lines in our research project was not human subject's research. MDA MB 435 variants were maintained using modified Eagle (MEM) medium (Mediatech Inc., Herndon, VA) supplemented with 5% fetal bovine serum (FBS) with the exception of 435-Brain 1 which were supplemented with 10% FBS, 5 mM L-glutamine and 5 mM penicillin-streptomycin at 37°C with 5% carbon dioxide. MCF 10A (non tumorigenic human epithelial) and HeLa (human epithelial) cells were purchased from ATCC (Manassas, VA). MCF 10A cells were maintained in Mammary Epithelial Cell Basal medium (MEBM) with the following supplements: 0.5 ml hydrocortisone, bovine pituitary extract (BPE); 2 ml gentamicin sulfate amphotericin-B (GA-1000); 0.5 ml human recombined epidermal growth factor in buffered BSA saline solution (rhEGF); and 0.5 ml human recombinant insulin. HeLa cells were maintained in high glucose Dulbecco modified Eagle (DMEM) medium (Mediatech Inc., Herndon, VA) supplemented with 10% fetal bovine serum (FBS), 5 mM L-glutamine, and 5 mM penicillin-streptomycin at 37°C with 5% carbon dioxide.

### Antibodies

Antibodies recognizing IRF-1 and STAT-1 were from Santa Cruz (Santa Cruz, CA). Antibodies recognizing histone H3, acetylated histone H3, histone H4, acetylated histone H4, and rabbit and mouse immunoglobulin G (IgG) isotype control antibodies were from Millipore (Lake Placid, NY). Antibodies recognizing histone 3 (trimethyl K27), histone 3 (acetyl K27), histone 3 (trimethyl K9), histone 3 (acetyl K18), CBP, and EZH2 antibodies were from Abcam (Cambridge, MA). Antibody recognizing EZH2 was from Millipore (Lake Placid, NY); antibody recognizing Tubulin was from Santa Cruz (Santa Cruz, CA); HRP conjugated mouse antibody was from Promega (Madison, WI); and HRP conjugated rabbit antibody was from Pierce (Rockland, IL).

### siRNA Constructs and Transient Transfections

Short interfering RNA (siRNA) duplexes predesigned against human EZH2 (Qiagen) were used for transient knockdown of EZH2. Cells were transfected with 1 ug of EZH2 specific siRNA or All Star scrambled sequence control (Qiagen) using RNAiFect transfection reagent (Qiagen) according to the manufacturer's instructions and were treated with IFN-γ (500 U/ml) as indicated. Cells were lysed in NP-40 lysis buffer (1 M Tris pH 8.0, 1 M KCl, 10% NP-40, 0.5 M EDTA, 5 M NaCl, 1 M DTT, dH_2_O) with EDTA free protease inhibitor (Roche) and analyzed by western blot for knockdown efficiency and specificity.

### Flow cytometry for MHC II cell surface expression

Cells were plated at a density of 7×10^5^, stimulated with IFN-γ (500 U/ml) as indicated, and were trypsinized, washed with PBS and resuspended in 100 µl of incubation buffer (0.5% bovine serum albumin in PBS). 10 µg Phycoerythrin (PE)-labeled anti-human *HLA-DR* (clone L243, Biolegend, San Diego, CA) or PE mouse IgG2a κ isotype control antibody (Biolegend) was added and the cell suspension was rotated at 4°C for 45 minutes. Following antibody incubation, cells were washed with PBS and fixed with 2% paraformaldehyde. MHC II cell surface expression was measured by FACS-Canto (Becton Dickinson, San Jose, CA) and analyzed using FlowJo. All samples were analyzed using 10,000 events per sample.

### Flow cytometry for surface expression of MHC II with EZH2 siRNA

Cells were plated at a density of 7×10^5^, transfected with 1 µg EZH2 specific or control siRNA (Qiagen), and twenty four hours post transfection, stimulated with IFN-γ (500 U/ml) as indicated. Following the stimulation cells were trypsinized, washed with PBS and 10% of the cell volume was lysed with 1% Nonidet P-40 lysis buffer and was analyzed by western blot for knockdown efficiency and specificity. The remaining cell volume was resuspended in 100 µl of incubation buffer and labeled as above.

### RNA Expression

Cells were plated at a density of 8×10^5^ cells and were stimulated as indicated with IFN-γ (500 U/ml). Cells were harvested and were subjected to RNA extraction with 1 ml of Trizol reagent (Invitrogen) as previously described [28], [43]. An Omniscript Reverse Transcription Kit (Qiagen) was used to generate cDNA from extracted RNA. Isolated DNA was analyzed by Q-PCR on an ABI prism 7900 (Applied Biosystems) using primers and probes for CIITApIII [44], CIITApIV, *HLA-DRA*, and GAPDH [28], [43]. Primer sequences for EZH2 were as follows: EZH2 sense TTCATGCAACACCCAACACT, EZH2 antisene GAGAGCAGCAGCAAACTCCT, and EZH2 probe FAM-TTACCAGCATTTGGAGGGAG-TAMRA. Values generated from Q-PCR reactions were calculated on the basis of standard curves generated.

### RNA expression in siRNA treated cells

Cells were plated at a density of 8×10^5^ cells, transfected with EZH2 specific or control siRNA, and twenty four hours later were stimulated as indicated with IFN-γ (500 U/ml). Cells were harvested and 10% of the cell volume was lysed with 1% Nonidet P-40 lysis buffer and subjected SDS PAGE. RNA was extracted and analyzed from the remaining fraction of cell volume as above.

### Chromatin Immunoprecipitation (ChIP)

ChIP assays were performed as previously described [27], [28], [43], [45]. Briefly, cells were plated at a cell density of 3×10^6^, stimulated with IFN-γ as indicated (500 U/ml), and were crosslinked with 1% formaldehyde for 10 minutes at room temperature. Crosslinking was stopped by the addition of 0.125 M glycine for five minutes at room temperature. Cells were lysed in SDS lysis buffer and were sonicated at constant pulse to generate an average of 500–750 bp sheared DNA. Sonicated lysates were precleared with salmon-sperm coated agarose beads (Upstate) and half of the lysate was immunoprecipitated with 5 µg of indicated antibody overnight at 4°C. The remaining half of the lysate was immunoprecipitated with isotype control antibody (Upstate). Following a two hour immunoprecipitation with 50 µl of salmon-sperm coated agarose beads, samples were washed for 3 minutes at 4°C with each of the following buffers: low salt buffer, high salt buffer, LiCl, and 1×TE and were eluted with SDS elution buffer [45]. Following elution, cross-links were reversed overnight with 5 M NaCl at 65°C and immunoprecipitated DNA was isolated using phenol∶chloroform∶isopropanol mix (Invitrogen) as per the manufacturer's instructions. Isolated DNA was analyzed by Q-PCR using previously published primers spanning the W-X-Y box of the *HLA-DRA* promoter and the IRF-E-GAS box of CIITApIV [27], [28], [43]. Values generated from Q-PCR reactions were calculated based on standard curves generated.

### Dual crosslinking Chromatin Immunoprecipitation (dChIP) Assay

Cells were plated at a density of 2.5×10^6^ cells and were stimulated as indicated with 500 U/ml IFN-γ. Cells were harvested and washed with PBS, resuspended in 10 ml of PBS (pH 8.0) containing 1 mM MgCl_2_ and 1 µM of Disuccinimidyl Glutarate (DSG) and were incubated at room temperature for 45 minutes as described above [29], [45]. Following crosslinking, 0.1 mM Tris pH 7.4 was added for five minutes at room temperature to block further cross-linking. Cells were washed in PBS, resuspended in 10 ml of PBS, and formaldehyde cross-linking was performed as above.

### Chromatin Immunoprecipitation with siRNA

ChIP siRNA assays were performed as previously described [27], [28]. Briefly, cells were plated at a density of 8×10^5^, transfected with 1 µg EZH2 specific or control siRNA (Qiagen), and stimulated as indicated with IFN-γ (500 U/ml). 10% of the cell volume was lysed with 1% Nonidet P-40 lysis buffer and was analyzed by western blot for knockdown efficiency and specificity. The remaining cell volume was crosslinked, lysed in SDS lysis buffer, and subjected to sonication and ChIP assay as above.

### DNA methylation analysis

MDA MB 435 breast cancer cells or HeLa cells were plated at a density of 7×10^5^, trypsinized, washed with PBS and re-suspended in 5 mL of PBS. Bisulfite treatment of DNA was performed using the EZ DNA methylation kit (Zymo Research Orange, CA) according to the manufacturer's instructions. Hot start touchdown PCR was performed in 1× PCR buffer, 0.25 mM dNTP, 0.5 µM forward and reverse primer, and 1.0 U Taq polymerase. Primers specific for CIITApIV CpG island region 2 [46] were used to amplify the desired segment and the PCR product was run on 1.5% agarose gel, gel extracted, and sequenced.

## Results

### Differential expression of MHC II by high and low metastatic variants of MDA MB 435 human breast carcinoma correlates with decreased expression of *HLA-DRA* mRNA

**Figure 1:**
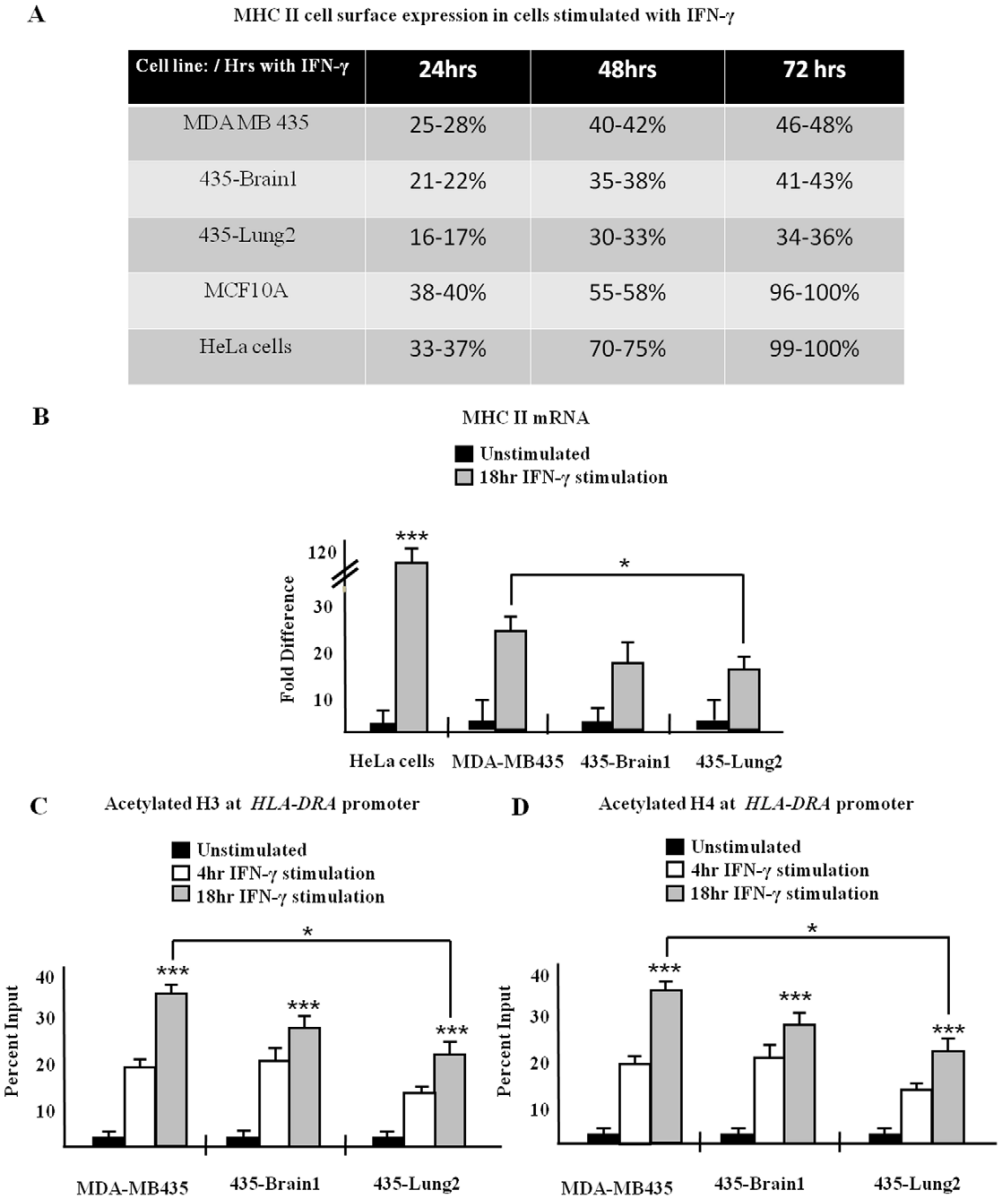
MHC II cell surface expression (A) and mRNA expression (B–C) is decreased in MDA MB 435 cells. (A) Cell surface expression of MHC II on MDA MB 435 variants, HeLa cells, and non metastatic breast MCF 10A cells. Cells were stimulated with IFN-γ as indicated, trypsinized, washed, and incubated with PE-labeled anti-human *HLA-DRA* antibody. Following antibody incubation, cells were fixed and PE cell surface staining was measured by FACS-Canto and analyzed using Flow Jo software. Results shown are the percent of MHC II positive events of four independent experiments. (B) Reduced expression of endogenous *HLA-DRA* mRNA in MDA MB 435 breast cancer cells compared to HeLa cells. Cells were left unstimulated or were stimulated with IFN-γ as indicated and levels of mRNA were measured by Q-PCR and were normalized to GAPDH mRNA. Results shown represent the mean ± SD of four independent experiments. (C–D) Levels of acetylated H3 (C) and acetylated H4 (D) at the *HLA-DRA* promoter. ChIP assays were carried out in cells stimulated as indicated with IFN-γ. Lysates were immunoprecipitated with control antibody, with antibody to acetylated H3 (C) or with antibody to acetylated H4 (D) and associated DNA was isolated and analyzed via real time Q-using primers spanning the W-X-Y box of the *HLA-DRA* promoter. Values for acetylated H3 (C) and acetylated H4 (D) IPs represent mean ± SEM of three independent experiments. Control IP values for (C) and (D) were 0.4±0.1*** P<0.0005, **P<0.005, *P<0.05.

Flow cytometry was used to determine cell surface expression of MHC II (versus mouse IgG2a κ isotype control, Fig. S1A) in unstained cells (data not shown), in three variants of MDA MB 435 human breast cancer cells, in HeLa cells, and in immortalized, but non-tumorigenic, epithelial breast MCF 10A cells in response to IFN-γ stimulation (Fig. 1A). Results shown are the percent of MHC II positive events. Following 24 hours of IFN-γ stimulation, average of 35% of HeLa cells were positive for surface expression of MHC II, and average of 40% of MCF 10A cells were MHC II positive. Following 24 hours of IFN-γ stimulation, on average 28% of MDA MB 435 cells, 22% of 435-Brain 1, and 16% of435-Lung 2 cells were positive for MHC II cell surface expression. Following 48 and 72 hours of cytokine stimulation, MDA MB 435 variants were, on average, 40% positive for cell surface MHC II, while HeLa and MCF 10A cells were nearly 100% MHC II positive. *HLA-DRA* mRNA levels in MDA MB 435 cells (Fig. 1B) demonstrate a significant 100 fold reduction in *HLA-DRA* message level when compared to HeLa cells and are further decreased in each of the MDA 435 cell variants (Fig. 1B). *HLA-DRA* mRNA levels were normalized to GAPDH mRNA which remained constant.

### Histone H3 and histone H4 are acetylated at the activated *HLA-DRA* promoter in MDA MB 435 cell variants

Transcription occurs within the highly ordered context of chromatin and an open chromatin structure is required for transcription initiation. To determine the availability of *HLA-DRA*, levels of acetylation on histone H3 and H4 were assayed in MDA MB 435 cell variants. Cells were left untreated or were stimulated with IFN-γ as indicated and were subjected to chromatin immunoprecipitation (ChIP) analyses. Lysates were immunoprecipitated with antibody against acetylated H3 (Fig. 1C) or acetylated H4 (Fig. 1D) and were analyzed via Q- PCR with primers and probe spanning the *HLA-DRA* promoter. Following IFN-γ stimulation, levels of acetylated H3 and H4 increase in all MDA MB 435 variants. However levels of acetylated H3 (Fig. 1C) and acetylated H4 (Fig. 1D) were significantly reduced in IFN-γ stimulated 435-Lung 2 cells when compared to the heterogeneous MDA MB 435 parental cell line. As previous studies demonstrate histone H3 lysine 18 (K18) is strongly acetylated at activated *HLA-DRA* [47], ChIP experiments were next performed to determine levels of *HLA-DRA* acetylation on H3K18 in MDA MB 435 variants. Cells were stimulated with IFN-γ as indicated, were subjected to ChIP analyses with antibody against acetylated H3K18, and were analyzed by Q-PCR with primers and probe spanning the *HLA-DRA* promoter. Levels of H3K18 acetylation were elevated with cytokine stimulation and no significant changes in H3K18 acetylation were observed between variants of MDA MB 435 cells or in comparison to HeLa cells (Fig. S2A). The histone acetyltransferase (HAT) CREB binding protein (CBP/p300) interacts with CIITA [16], [48] and is known to acetylate both H3 and H4 at the *HLA-DRA* promoter and to enhance binding of CIITA to the MHC II enhanceosome complex [49]. ChIP analyses indicate recruitment of CBP to *HLA-DRA* upon cytokine stimulation in each of the MDA MB 435 variants and in HeLa cells (Fig. S2B). Together, these data indicate that loss of inducible *HLA-DRA* promoter acetylation in MDA MB 435 cells is not responsible for the inability of these cells to inducibly express maximum levels of cells surface MHC II.

### Silencing modifications decrease at the activated *HLA-DRA* promoter in MDA MB 435 cell variants

In order to determine patterns of histone trimethylation of histone H3 lysine 9 (H3K9me3) and of histone H3 lysine 27 (H3K27me3) at the *HLA-DRA* promoter, HeLa cells and MDA MB 435 cell variants were stimulated with IFN-γ and ChIP analysis was performed. Levels of H3K9me3 and H3K27me3 were significantly reduced at the *HLA-DRA* promoter within 4 hrs of cytokine stimulation and were eliminated 18 hrs post IFN-γ stimulation in each of the three variants of MDA MB 435 cells and in HeLa cells (Fig. S2C–D). Together these data indicate sustained histone methylation at the *HLA-DRA* promoter is not responsible for suppressed cell surface expression of MHC II in cytokine stimulated MDA MB 435 cell variants.

Acetylation of H3K27 (H3K27ac) is catalyzed by CBP and is associated with multiple active mammalian genes. To determine levels of H3K27ac on the *HLA-DRA* promoter in MDA MB 435 cells and in HeLa cells, we performed ChIP assays with antibodies recognizing H3K27ac. Levels of H3K27ac on *HLA-DRA* increase significantly with cytokine stimulation in each of the three variants of MDA MB 435 cells with levels comparable to H3K27ac in HeLa cells (Fig. S2E); further indication the *HLA-DRA* promoter is opened and available upon IFN-γ stimulation.

### Despite suppression of CIITA mRNA in MDA MB 435 variants, histone H3 and histone H4 are inducibly acetylated at CIITA pIV

**Figure 2:**
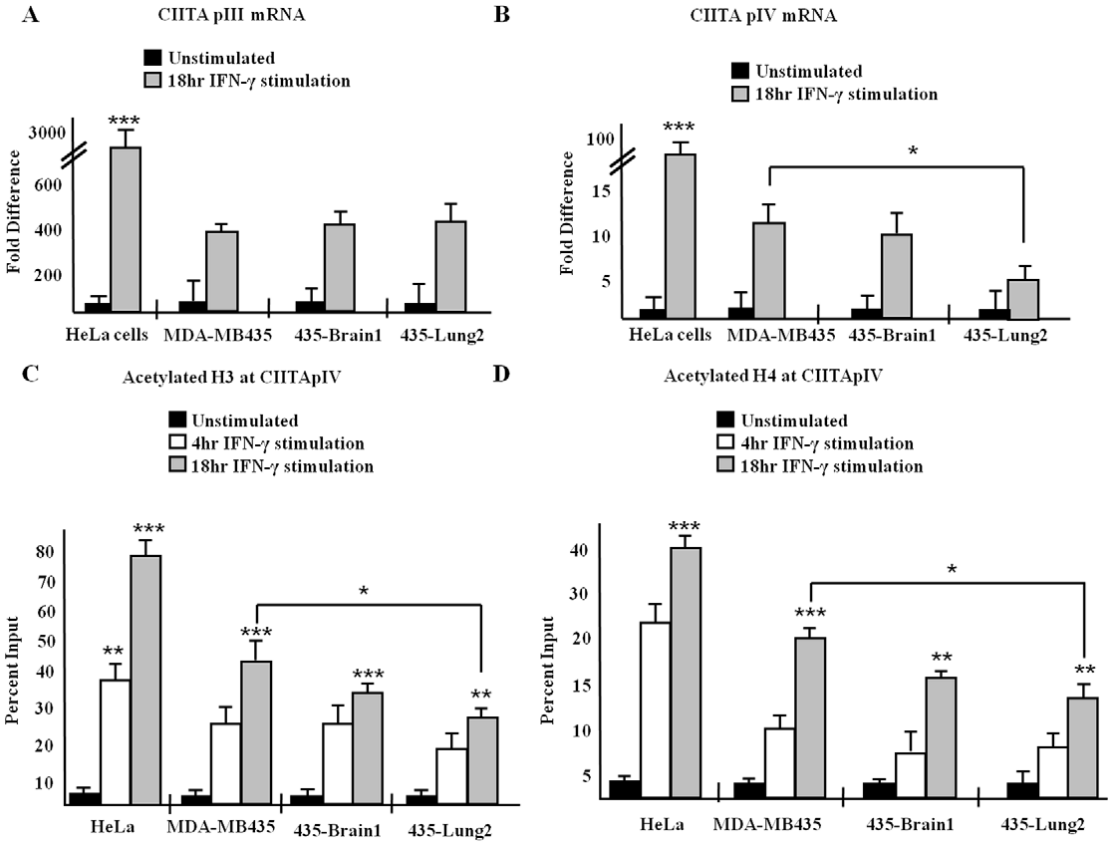
Decreased levels of CIITA isoform III and isoform IV mRNA correlates with low levels of acetylation. (A–B) HeLa and MDA MB 435, 435-Brain 1, and 435-Lung 2 cells were treated as indicated with IFN-γ. Levels of CIITApIII mRNA (A) and CIITApIV mRNA (B) were measured by Q-PCR. Results shown represent the mean ± SD of four independent experiments. (C–D) Levels of acetylated H3 (C) and acetylated H4 (D) at CIITApIV in MDA MB 435 and HeLa cells. ChIP assays were carried out in cells stimulated as indicated with IFN-γ. Lysates were IP with control antibody, with antibody to acetylated H3, (C) or with antibody to acetylated H4 (D) and associated DNA was isolated and analyzed via Q-PCR using primers spanning the IRF-E-GAS box of CIITApIV. Values for control IPs and acetylated H3 (C) and acetylated H4 (D) IPs represent the mean ± SEM of three independent experiments. Control IP values for (C) and (D) were 0.6±0.5 *** P<0.0005, **P<0.005, *P<0.05.

CIITA expression and subsequent binding to the *HLA-DRA* promoter is essential for transcriptional activation of *HLA-DRA* genes. Four different promoters have been described to regulate transcription of CIITA, each being characterized by a different first exon [25]. As promoters III and IV both drive CIITA expression following IFN-γ stimulation, we determined the relative expression of CIITA isoform III and isoform IV mRNA in stimulated HeLa cells and in each of the three MDA MB 435 variants. Cells were stimulated with IFN-γ as indicated and analyzed via Q-PCR using primers specific for CIITApIII (Fig. 2A) and for CIITApIV (Fig. 2B). In comparison to significant increases in CIITApIII and pIV mRNA expression in HeLa cells in response to IFN-γ stimulation, both CIITApIII and pIV expression levels are suppressed in each variant of MDA MB 435 cells (Fig. 2A–B).

**Figure 3:**
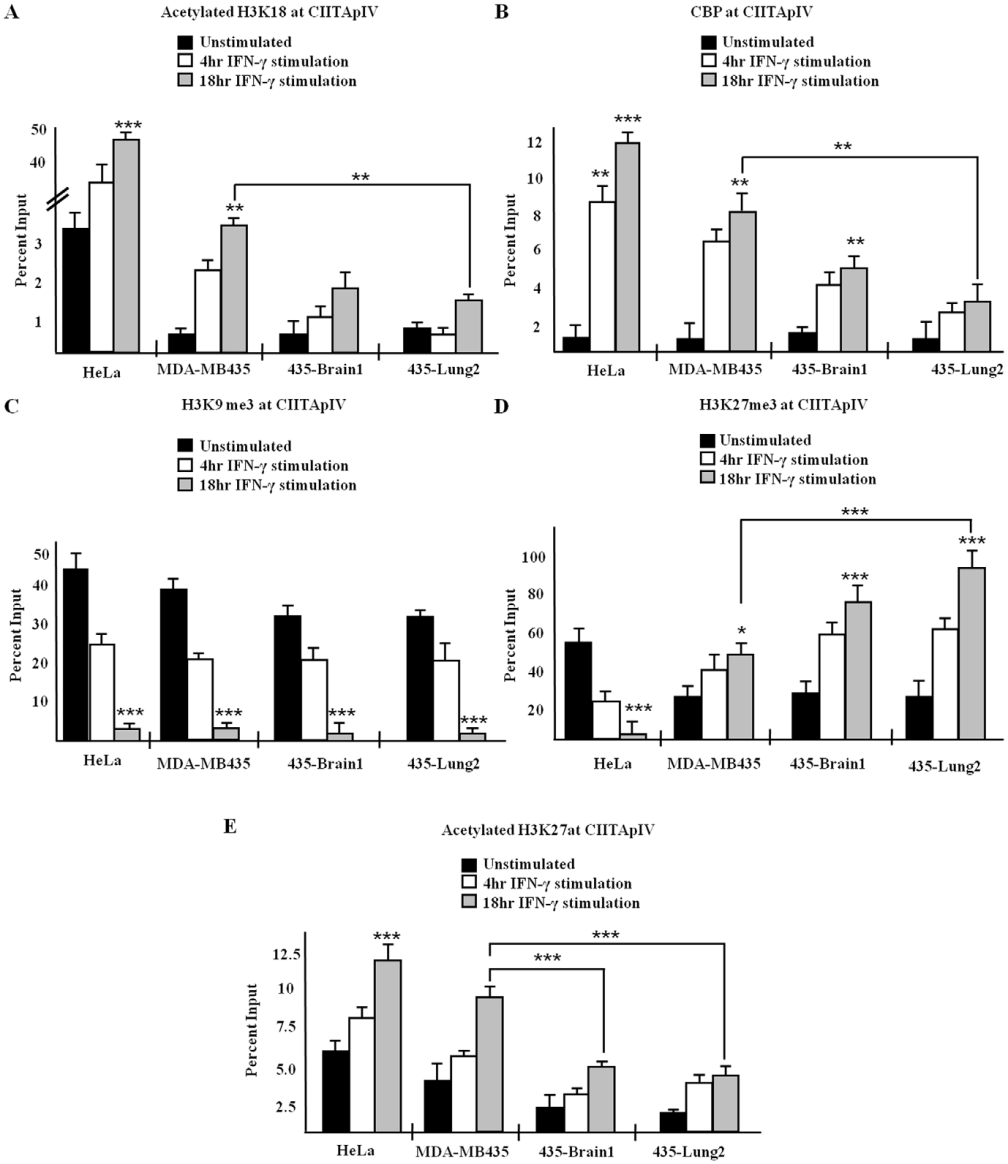
Decreased levels of CIITApIV H3K18ac, H3K27ac and CBP correlate with significantly elevated levels of H3K27me3 in metastatic breast cancer cells. (A–E) Levels of acetylated H3K18, CBP, H3K9me3, H3K27ac, and H3K27me3 at CIITApIV. ChIP assays were carried out in MDA MB 435, 435-Brain 1, 435-Lung 2, and HeLa cells stimulated as indicated with IFN-γ. Lysates were IP with control antibody or antibody to indicated proteins and associated DNA was isolated and analyzed as above. Values shown represent mean ± SEM of three to five independent experiments. Control IP values for (A–E) were 1.3±0.7 ***P<0.0005, **P<0.005.

Our observations of significant decreases in CIITApIV transcripts between MDA MB 435 variants (Fig. 2B) led us to next focus our analysis on the levels of global histone acetylation at CIITApIV using ChIP assays and antibodies against acetylated H3 and acetylated H4. Q-PCR analysis indicated that levels of acetylated H3 (Fig. 2C) and of acetylated histone H4 (Fig. 2D) at CIITApIV decrease between MDA MB 435 variants upon stimulation with IFN-γ. Additionally, levels of CIITApIV H3 and H4 acetylation in HeLa cells are significantly more robust than those in the MDA MB 435 cell variants (Fig. 2C–D). To analyze levels of acetylated H3K18 and association of the HAT CBP at CIITApIV in the MDA MB 435 variants, cells were left untreated or were stimulated with IFN-γ as indicated, subjected to immunoprecipitation with antibody to acetylated H3K18 (Fig. 3A) or CBP (Fig. 3B), and were analyzed via Q-PCR with primers and probes spanning the CIITApIV proximal promoter. Total levels of acetylated H3K18 and CBP at CIITApIV in 435-Brain 1 and 435-Lung2 cells significantly decreased upon cytokine stimulation in comparison with the heterogeneous parental MDA MB 435 cells. Levels of inducible H3K18 acetylation and levels of CBP binding at CIITApIV were both lower in each of the MDA MB 435 variants in comparison to HeLa cells (Fig. 3A–B). As total levels of CBP remain unchanged between MDA MB 435 variants (Fig. S3C), CBP binding, not expression, likely accounts for decreased histone acetylation within the variants.

### CIITApIV is specifically and inducibly hypermethylated at CIITApIV in MDA MB 435 cell variants

To determine CIITApIV levels of H3 lysine 9 and lysine 27 methylation and levels of lysine 27 acetylation in MDA MB 435 cell variants, ChIP experiments were performed using antibodies against H3K9me3, H3K27me3, and H3K27ac. Q-PCR analysis indicated elevated basal levels of H3K9me3 at CIITApIV that significantly decrease upon stimulation with IFN-γ in the MDA MB 435 variants and in HeLa cells (Fig. 3C). Basal levels of CIITApIV H3K27me3 were observed in MDA MB 435 cell variants; however, following IFN-γ stimulation, CIITApIV levels of H3K27me3 significantly, and unexpectedly, increased correlative with increasing metastatic potential of MDA MB 435 cell variants (Fig. 3D). The inducible hypermethylation of lysine 27 observed at CIITApIV is cell line specific as ChIP assays performed in HeLa cells demonstrate an opposite trend where elevated levels of CIITApIV H3K27me3 in unstimulated cells significantly decrease upon IFN-γ stimulation (Fig. 3D). We further observed that maximum levels of cytokine induced H3K27ac decrease between the MDA MB 435 variants and when these variants are compared to HeLa (Fig. 3E).

To determine if epigenetic alterations at CIITApIV are sequence specific, ChIP assays were performed to detect levels of H3K27me3, H3K9me3, H3K27ac, and H3K18ac at the GAPDH promoter (Fig. S1B). Low levels of methylation and high levels of acetylation were observed at the GAPDH promoter that were unchanged by IFN-γ stimulation and were not significantly different between MDA MB 435 cell variants. Gains in H3K27methylation at CIITApIV in the MDA MB 435 cell variants are not indicative of increases in histone H3 or histone H4 as ChIP assays demonstrate no significant changes in the level of H3 (Fig. 3A) or H4 (supplementary Fig. S3B) in any of the MDA MB 435 cell variants. In sum, these data indicate elevated levels of inducible H3K27me3 at CIITApIV are likely responsible for the increasingly suppressed levels of CIITA mRNA in MDA MB 435 cell variants.

### IFN-γ inducible recruitment of STAT-1 and IRF-1 to CIITApIV is diminished in MDA MB 435 cell variants

The transcription factors STAT-1 and IRF-1 are both required for CIITApIV transcription in response to IFN-γ stimulation [25]. To determine if the lack of CIITA mRNA in MDA MB 435 cell variants was due to reduced expression of STAT-1 and IRF-1, Western blot analyses were performed. Levels of STAT-1 and IRF-1 remain consistent in the MDA MB 435 variants (Fig. S3C), indicating both STAT-1 and IRF-1 are expressed and available for CIITApIV binding. Levels of phosphorylated STAT-1 (pSTAT-1) are similarly induced in the MDA MB 435 variants, indicating activation of the JAK-STAT pathway is unaffected (Fig. S3C).

**Figure 4:**
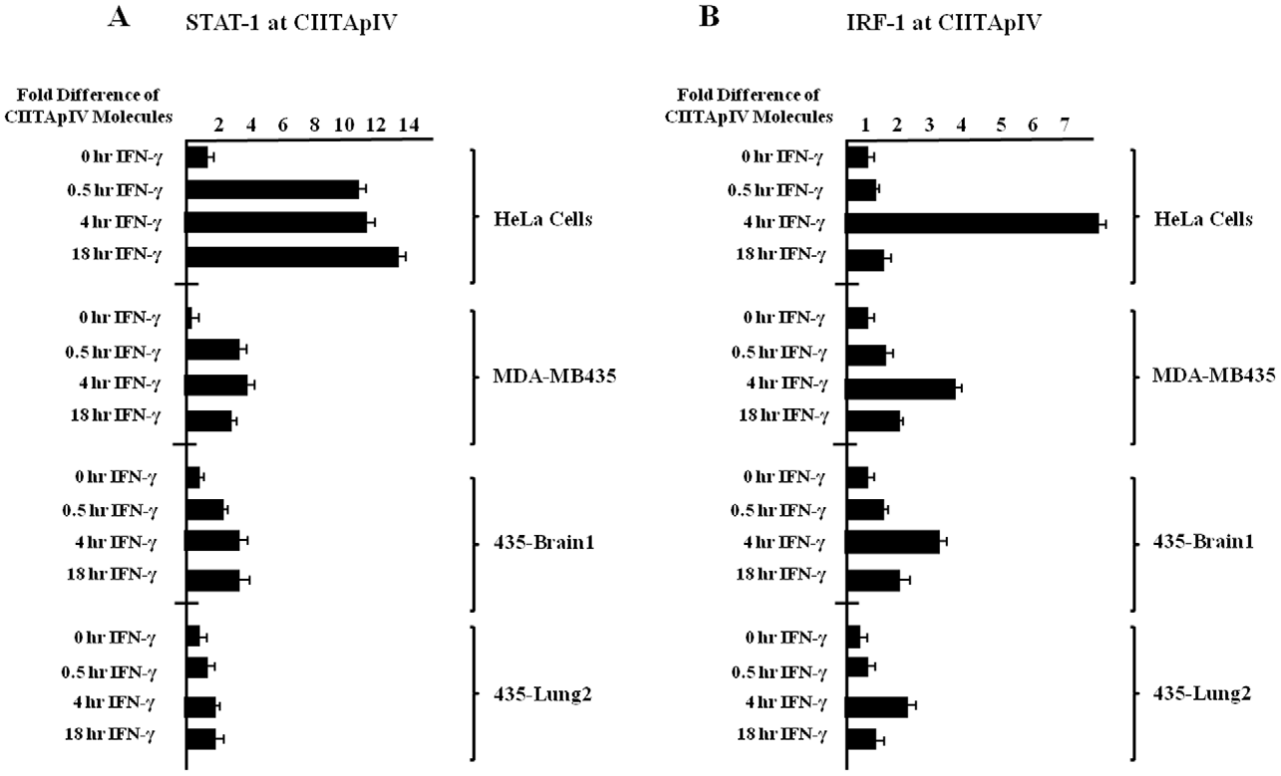
Decreased recruitment of STAT-1 and IRF-1 to CIITApIV in metastatic breast cancer cells. (A–B) ChIP assays were carried out in MDA MB 435 variants and HeLa cells stimulated as indicated with IFN-γ. Lysates were IP with control antibody, with antibody to STAT-1 (A), or with antibody to IRF-1 (B), and associated DNA was analyzed as above. IP values shown are presented as increases in CIITApIV promoter DNA relative to unstimulated STAT-1 (A) and IRF-1 (B) IP samples. Values for control IPs, STAT-1 IP's, and IRF-1 IPs represent mean ± SEM of three independent experiments. Control IP values for (A) and (B) were 0.2±0.3.

An open chromatin confirmation is required for the initiation of transcription. Data in Fig. 3 indicate that CIITApIV is hypermethylated and in a closed confirmation in cytokine stimulated MDA MB 435 cell variants. To confirm the closed status of chromatin at CIITApIV, promoter recruitment of STAT-1 and IRF-1 was analyzed by ChIP assays in MDA MB 435 variants and in HeLa cells. Cells were stimulated with IFN-γ as indicated and were subjected to immunoprecipitation with antibody recognizing STAT-1 or IRF-1. ChIP data indicate low level recruitment of STAT-1 and IRF-1 to CIITApIV in each of the MDA MB 435 variants with minimal increases in binding following IFN-γ stimulation (Fig. 4A–B). Levels of STAT-1 and IRF-1 binding to CIITApIV were significantly diminished in comparison to STAT-1 and IRF-1 binding to CIITApIV in HeLa cells (Fig. 4A–B).

### Binding of the histone methyltransferase EZH2 to CIITApIV is significantly and specifically increased in MDA MB 435 cell variants

**Figure 5:**
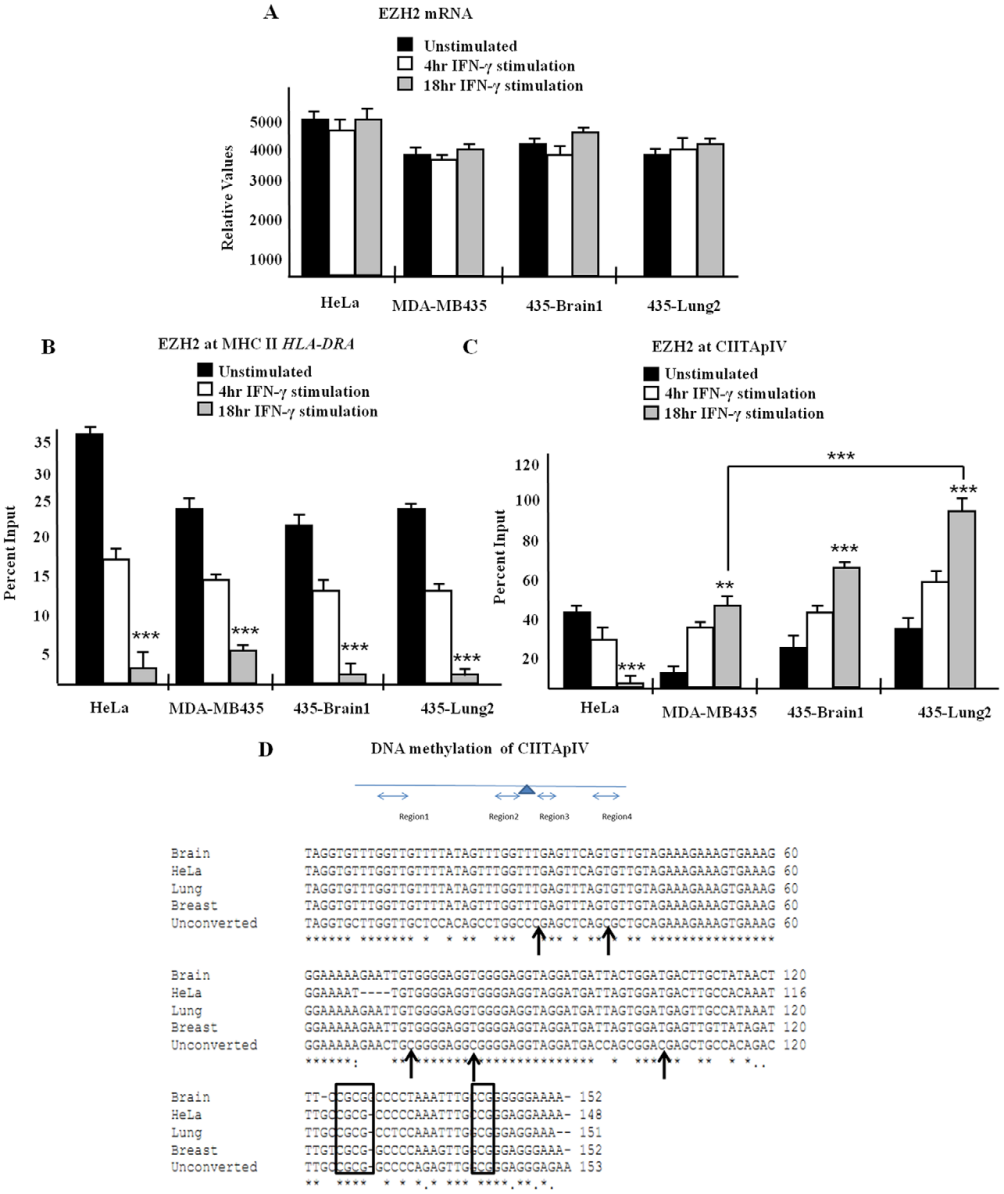
IFN-γ stimulation results in specific and significantly increased binding of EZH2 to CIITApIV in MDA MB 435 cells. (A) EZH2 mRNA expression. MDA MB 435, 435-Brain 1, 435-Lung 2, and HeLa cells were stimulated as indicated with IFN-γ. Cell lysates were analyzed for levels of EZH2 mRNA as above. Q-PCR was performed in triplicate and results represent the mean ± SD of three independent experiments. (B–C) Levels of EZH2 at *HLA-DRA* (B) and CIITApIV (C) promoters post cytokine stimulation. Double crosslinking ChIP assays were carried out in cells stimulated as indicated with IFN-γ. Lysates were IP with control antibody or with antibody to EZH2 and associated DNA was isolated and analyzed via Q-PCR using primers spanning the W-X-Y box of *HLA-DRA* (B) and using primers spanning the IRF-E-GAS box of CIITApIV (C). Values for control IPs and for and EZH2 IPs represent mean ± SEM of three independent experiments. Control IP values for (A) and (B) were 2.1±0.5 ***P<0.0005. (D) DNA methylation analyses of CIITApIV. Sequence analysis of CIITApIV region 2 of MDA MB 435 cells and HeLa cells indicates no differences in CpG island methylation. Primers spanning region 2 of CIITApIV were used to amplify DNA and purified PCR product was sequenced and aligned with cells lines. Arrows indicate unmethylated CpG sites. Boxes indicate methylated CpG sites conserved between MDA MB 435 cells and HeLa cells.

Histone methyltransferases (HMTs) are chromatin remodeling enzymes that add one, two, or three methyl groups to lysine residues on histones [50]. We have recently demonstrated the HMT enhancer of zeste homolog 2 (EZH2), a known regulator H3K9me3 and H3K27me3 [36], [51], to be a critical regulator of IFN-γ inducible transcription from CIITApIV [29]. Initial analyses confirmed that each of the MDA MB 435 variants expresses similar levels of EZH2 mRNA (Fig. 5A) and EZH2 protein (Fig. S3E). To determine if EZH2 aberrantly binds CIITApIV in the MDA MB 435 variants and HeLa cells, ChIP assays were performed. Cells were stimulated with IFN-γ as indicated and were subjected to immunoprecipitation with antibody against EZH2. Chromatin immunoprecipitation showed comparable EZH2 binding to *HLA-DRA* (Fig. 5B) and to CIITApIV (Fig. 5C) in unstimulated cells (note Y axis differences). Four hours post cytokine stimulation, EZH2 occupancy decreases at *HLA-DRA* promoter and reaches baseline binding 18 hours following cytokine stimulation (Fig. 5B). Striking differences in EZH2 binding patterns were observed at CIITApIV in the MDA MB 435 variants. In unstimulated cells, EZH2 binds to CIITApIV at levels similar to that of *HLA-DRA*. However, upon cytokine stimulation, EZH2 binding increases in each variant of MDA MB 435 cells, at both four and 18 hours post IFN-γ stimulation (Fig. 5C). By comparison, in HeLa cells, patterns of EZH2 binding to CIITApIV (Fig. 5C) are similar to binding of EZH2 at *HLA-DRA* (Fig. 5B).

#### Analysis of CIITApIV CpG islands indicates no differences in DNA methylation within the variants of the MDA MB 435 cells

In trophoblasts, expression of CIITA is blocked by CIITApIV start site proximal DNA methylation [52] and DNA methylation at regions 2 and 3 of the 5′ CIITApIV CpG island has been detected in colorectal and gastric cancers which lack CIITA expression [46]. Previous studies indicate 435-Lung2 cells treated with 5-aza CR, an inhibitor of DNA methylation, restore expression of CIITA mRNA and MHC II protein synthesis [42]. To more fully address roles for promoter proximal DNA methylation in suppression of CIITApIV in MDA MB 435 variants, we used four primer sets and bisulfate restriction analysis to analyze DNA methylation levels at regions 2 and 3 of the 5′ CIITApIV CpG island in each of the MDA MB 435 variants (Fig. 5D) [46]. No differences in methylated (boxed regions) or unmethylated (arrowed regions) DNA were detected between variants of MDA MB 435 cells, suggesting reduced CIITA expression in the variants of MDA MB 435 are not due to changes in DNA methylation.

### Knockdown of EZH2 significantly reduces H3K27me3 at CIITApIV in the MDA MB 435 variants

**Figure 6:**
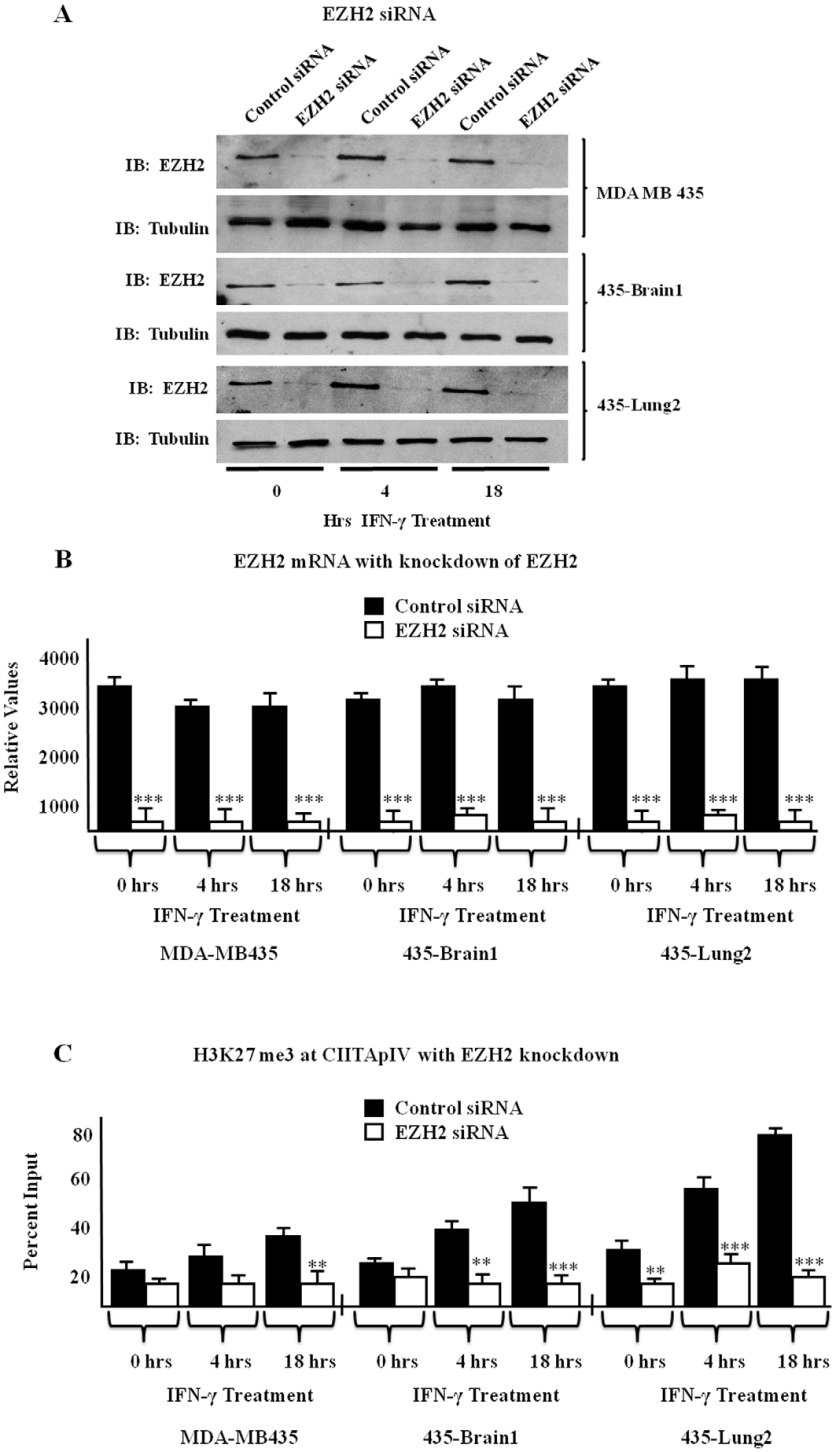
EZH2 knockdown decreases CIITApIV histone H3K27 trimethylation. (A–B) EZH2 knockdown is efficient and specific. MDA MB 435, 435-Brain 1, and 435-Lung 2 cells were transfected with either EZH2 specific siRNA or with control siRNA. 10% of lysates were subjected to immunoblot (IB) for EZH2 and tubulin. Results reported are representative data from three independent experiments. The remaining lysates were subjected to EZH2 mRNA isolation and quantitation by Q-PCR. Q-PCR was performed in triplicate and results shown represent the mean ± SD of three independent experiments. (C) Levels of trimethylated H3K27 in breast cancer cells. ChIP assays were carried out in MDA MB 435 variants transfected with either EZH2 siRNA or control siRNA and stimulated as indicated with IFN-γ. Lysates were IP with control antibody or with antibody to H3K27me3 and associated DNA was isolated and analyzed as above via Q-PCR using primers spanning the IRF-E-GAS box of CIITApIV. Values shown represent mean ± SEM of three independent experiments. Control IP values were 1.2±0.6 ***P<0.0005, **P<0.005.

To further investigate roles for EZH2 in the suppression of CIITApIV in the MDA MB 435 variants, we utilized siRNA duplexes to specifically knock down expression of EZH2 and performed ChIP assays to detect levels of H3K27me3 at CIITApIV. siRNA mediated knockdown of EZH2 resulted in specific decreases in EZH2 protein expression (Fig. 6A). To further determine efficiency of the siRNA duplexes, EZH2 mRNA levels were tested in each of the MDA MB 435 variants (Fig. 6B). Cells treated with EZH2 specific siRNA (white bars) showed significant reductions in EZH2 mRNA levels when compared to cells treated with control siRNA (black bars). To determine levels of H3K27me3 at CIITApIV in the EZH2 siRNA treated MDA MB 435 cell variants, ChIP assays were performed. In cells treated with control siRNA, levels of H3K27me3 increase at CIITApIV upon IFN-γ stimulation (Fig.6C, black bars). However, when specific siRNA was used to knockdown EZH2, significant decreases in CIITApIV H3K27me3 were observed in each of the MDA MB 435 variants upon IFN-γ treatment (Fig. 6C, white bars). These data suggest EZH2 is responsible for the elevated levels of CIITApIV H3K27me3 in the variants of MDA MB 435.

### Knocking down EZH2 restores suppressed levels of CIITApIV and *HLA-DRA* mRNA as well as cell surface expression of MHC II in each of the variants of MDA MB 435

**Figure 7:**
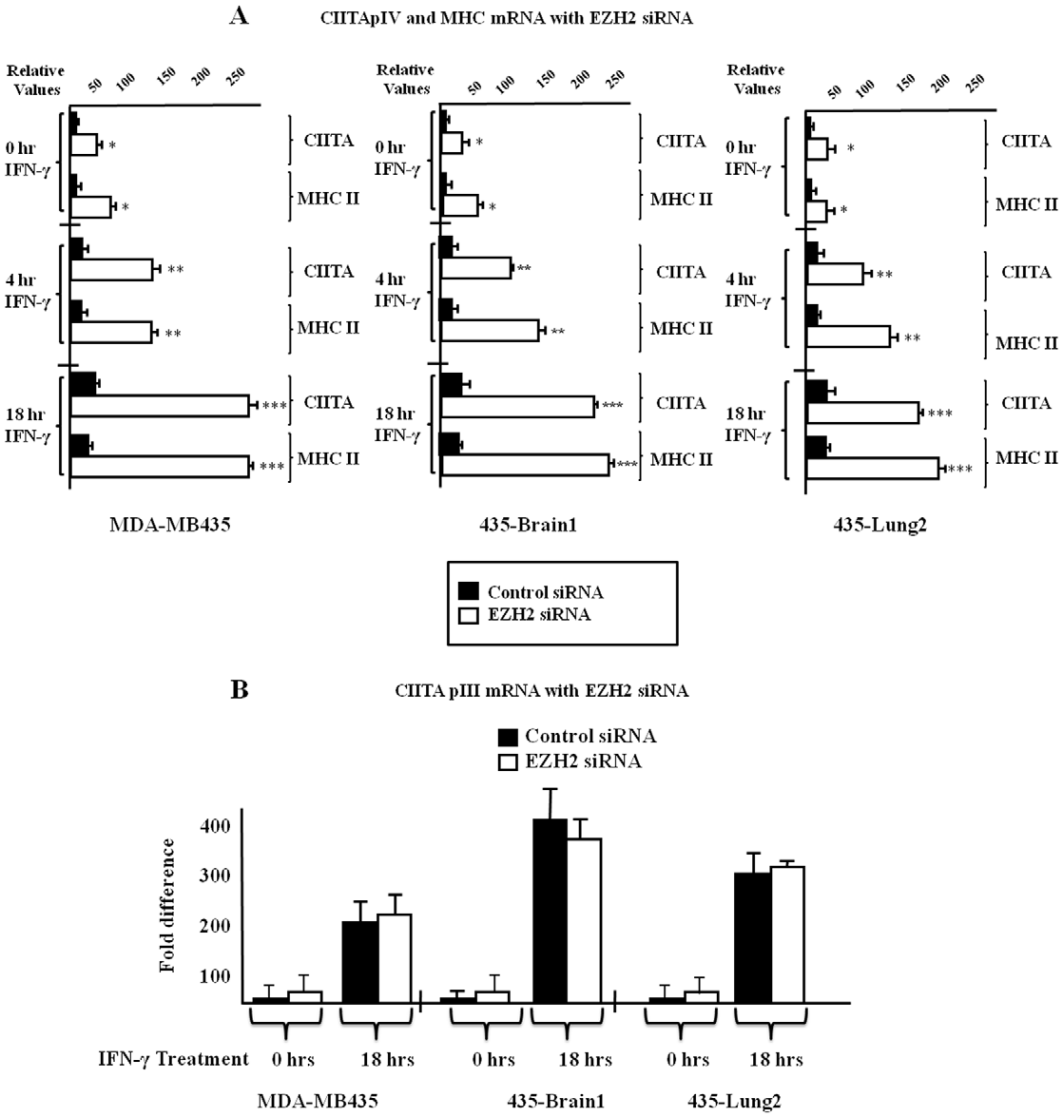
EZH2 knockdown significantly increases mRNA levels of CIITApIV and *HLA-DRA* but not CIITApIII in MDA-MB 435 variants. (A–B) MDA MB 435 variants were transfected with either EZH2 specific siRNA or with control siRNA and were stimulated as indicated with IFN-γ. Levels of *HLA-DRA* mRNA and CIITApIV mRNA (A) and levels of CIITApIII mRNA (B) were measured by Q-PCR and were normalized to levels of GAPDH mRNA. Q-PCR was performed in triplicate and results shown represent the mean ± SD of three independent experiments. ***P<0.0005, **P<0.005, *P<0.05.

To determine if decreased expression of EZH2 and a resulting decrease in CIITApIV H3K27me3 can reconstitute CIITA and *HLA-DRA* gene expression in the MDA MB 435 variants, mRNA experiments were performed. Cells were transfected with EZH2 specific or control siRNA and were stimulated with IFN-γ as indicated. Following stimulation cells were lysed and CIITApIV and *HLA-DRA* mRNA levels were quantified via Q-PCR. Both CIITA and *HLA-DRA* mRNA levels are significantly elevated in cells treated with EZH2 specific siRNA (Fig. 7A, white bars) when compared to cells treated with control siRNA (Fig. 7A, black bars). Significantly, decreased expression of EZH2 increases both CIITA and *HLA-DRA* message levels in each of the MDA MB 435 variants, even in the absence of IFN-γ stimulation. We further investigated roles for EZH2 in restoring expression of CIITApIII by using EZH2 specific siRNA to knockdown EZH2 expression, followed by analysis of CIITApIII mRNA. Both EZH2 and control siRNA treated cells demonstrated similar levels of CIITApIII mRNA with and without IFN-γ stimulation, indicating roles for EZH2 in suppressing CIITA expression are promoter specific ( Fig. 7B). Flow cytometry was used to determine if MDA MB 435 variants expressed increased cell surface MHC II in response to IFN-γ stimulation when treated with EZH2 specific siRNA. Levels of MHC II expression in the MDA MB 435 parental cells line, the 435-Brain1 variant, and the 435-Lung2 variant, were all enhanced in EZH2 siRNA treated samples when compared to cells transfected with control siRNA (Fig. S4). Together, these studies suggest hypermethylation of histone tails silences CIITApIV gene expression in MDA MB 435 variants.

## Discussion

Our study correlates decreased expression of MHC II in breast cancer cells with epigenetic suppression of CIITApIV by the histone methyltransferase EZH2. In variants of MDA MB 435 cells, metastatic potential negatively correlates with expression of MHC II and with active transcription of the MHC II master regulator, CIITA. We show here that silencing of MHC II molecules in highly metastatic breast cancer cells is associated with distinct epigenetic modifications targeted specifically to the chromatin of CIITApIV. In these cells, transactivation from CIITApIV is actively suppressed by increased binding of the histone methyltransferase EZH2 and by increased H3K27me3, even in the presence of the inflammatory cytokine IFN-γ. These findings are supported by our recent observation that EZH2 is a master regulator of CIITA and indicate promoter specific roles for EZH2 [29].

Tumors which best avoid immune recognition are an increased risk for metastasis and tumor related mortality. Tumors of the breast are no exception to this rule with the most common sites for breast cancer metastasis being the lung, liver, bone, brain, and auxiliary lymph nodes [53]. To achieve metastatic ability, tumor cells specifically alter immune gene expression patterns in order to escape host immune surveillance. Multiple tumor types, including colorectal and gastric tumors [46], trophoblastic tumors [52], T cell leukemias, and developmental tumors [54], accomplish specific decreases in MHC II expression by silencing IFN-γ inducible CIITApIV [55]. While decreased MHC cell surface expression is considered to be an important factor in predicting tumor metastasis and patient prognosis, the mechanism of MHC II suppression has remained unknown [56], [57], [58]. Decreased expression of MHC II has been observed in metastatic breast tumors and in multiple breast cancer cell lines, indicating that suppression of MHC II may have causative, rather than correlative, relationships with immune evasion and with metastatic potential in breast tumors [39], [59], [60], [61], [62], [63]. We provide evidence that expression of MHC II genes correlates with the ability of breast cancer cells to metastasize. The more metastatic the MDA breast cancer cell line, the less MHC II is induced in the presence of IFN-γ stimulation. In these cell lines, decreases in IFN-γ inducible transcript levels of the MHC II master regulator CIITA are specifically associated with the silencing modification H3K27me3 at CIITApIV. Notably, acetylation levels were not significantly impacted in poorly or highly metastatic MDA variants, suggesting the dominance of H3K27me3 in the IFN-γ induced chromatin dynamics at CIITApIV. The ramifications of our study to tumors of the breast are clear and support previous findings regarding suppression of CIITA in breast cancer [42]: breast tumors may increase metastatic properties by epigenetically targeting CIITApIV resulting in suppression of cell surface expression of MHC II. As MHC cell surface expression is indispensable for T cell interaction and the presentation of tumor derived peptides to helper T cells [8], [9], breast cancer cells likely down regulate MHC II genes to avoid immune recognition. Silencing of MHC II molecules in metastatic cell lines is associated with dysregulated epigenetic modifications at the proximal promoter of CIITApIV. Significant decreases in CIITA transcript levels, even in cells stimulated with IFN-γ, resulted in both decreased *HLA-DRA* transcripts and in decreased cell surface expression of MHC II. Expression of MHC II genes correlates with metastatic ability of the MDA MB 435 variants as indicated by baseline expression of MHC II in the highly metastatic 435-Lung2 variant. The lack of transactivation from CIITApIV is not due to defective IFN-γ signaling as pSTAT-1 levels are unchanged between the MDA MB 435 variants. The requisite CIITApIV transcription factors STAT-1 and IRF-1 are expressed in the MDA MB 435 variants, but exhibit limited capability to bind CIITApIV, even in the presence of IFN-γ stimulation. Decreases in CIITA transcript levels correlate with elevated H3K27me3 at CIITApIV, while neither H3K9me3 nor H3 or H4 acetylation demonstrate significant alterations in the MBD MB 435 variants. Levels of activating H3K27ac are also significantly decreased at CIITApIV, and inversely correlate with high levels of H3K27me3. These observations underline the dominance of H3K27me3 in regulating the transcriptional status of CIITApIV chromatin.

As the catalytic subunit of PRC2, EZH2 adds three methyl groups to lysine 27 resulting in chromatin condensation. Converse to frequent observations of decreased MHC II expression in tumors are observations of overexpression of EZH2 in diverse tumors types including prostate, breast and bladder cancers and links to enhanced tumor cell proliferation [64], [65], [66]. Elevated expression of EZH2 correlates with metastatic tumor growth and with clinically aggressive behaviors in prostate and breast cancer [67], have been recognized as a negative prognostic marker for a number of breast and prostate cancer patients [68], [69], and are associated with poor outcomes to tamoxifen therapy in advanced breast cancer patients [70]. Our observations add to this growing field by indicating that dysregulated promoter recruitment of EZH2 to CIITApIV also occurs in MDAMB 435 variants and further indicates EZH2 as a primary culprit in metastatic tumor growth. We demonstrate here significant levels of EZH2 and H3K27me3 at CIITApIV in the 435-Lung2 cells, which have been previously characterized as highly metastatic [42]. Most striking is the novel observation that decreased expression of EZH2 results in constitutive expression of CIITApIV and *HLA-DRA* transcripts in unstimulated tumor cells; these results provide increased evidence for EZH2 as a target for anti-tumor immunotherapies and provide additional mechanistic links to roles for EZH2 in tumor cell metastasis.

Our results support a hypothesis that hypermethylation of histone tails by EZH2 but not DNA methylation represses CIITApIV gene expression in metastatic variants of MDA MB 435 cells. It is noteworthy that the master regulator CIITA is in turn controlled by its own master regulator, EZH2. One possibility is that critical CIITA silencing is the specific result of increased promoter occupancy and IFN-γ dependent recruitment of EZH2. These observations are in agreement with previous findings that overexpression of CIITA in MDA MB 435 variants blocks lung metastasis [71], and suggest EZH2 is a pivotal and specific contributor to CIITApIV silencing. While EZH2 has been demonstrated to interact with DNA methyltransferases and EZH2 binding has been shown to be required for DNA methylation of EZH2 target promoters [72], our data indicate no differences in the CpG methylation of a previously characterized promoter region of CIITApIV. Better understanding of the mechanisms responsible for decreased expression of MHC II in metastatic breast cancer cells will enable development of novel ways of enhancing tumor recognition and eradication by the immune system.

## Supporting Information

Figure S1 **Isotype control staining of variants of MDA-MB 435, HeLa, and MCF 10A, and levels of H3K27me3, H3K9me3, H3K18ac, and H3K27ac at the GAPDH proximal promoter, are unaffected by cytokine stimulation in MDA MB 435, 435-Brain 1, and 435-Lung 2 cells.** (A) MDA MB 435, 435-Brain 1, 435-Lung 2 cells, HeLa cells, and MCF 10A cells were stimulated with IFN-γ as indicated. Post stimulation cells were trypsinized, washed, and incubated with PE-labeled mouse control IgG. Following antibody incubation, cells were fixed and PE cell surface staining was measured by FACS-Canto. Results shown are representative of three independent experiments. (B) Cells were stimulated as indicated and were subjected to ChIP assay as above. Lysates were IP with isotype control antibodies or antibodies against indicated histone modification; associated DNA was isolated and analyzed via Q-PCR using primers spanning the GAPDH proximal promoter. Values shown represent mean ± SEM of two independent experiments. Average control IP values were 350±200
(TIF)

Figure S2 **IFN-γ induced levels of H3K18ac, H3K27ac, CBP, H3K9me3, and H3K27me3 at the** ***HLA-DRA*** **promoter in HeLa cells and MDA MB 435 variants.** (A–E) ChIP assays were carried out in HeLa cells, MDA MB 435 breast cancer cells, 435-Brain 1 cells, and 435-Lung2 cells stimulated as indicated with IFN-γ as described above. DNA was isolated and analyzed via Q-PCR using primers spanning the W-X-Y box of the *HLA-DRA* promoter. Values represent mean ± SEM of two independent experiments for HeLa and four independent experiments for MDA MB 435 variants. Average control IP values were 0.9±0.7
(TIF)

Figure S3 **Levels of H3 and H4 remain unchanged in HeLa cells and in MDA MB 435 variants stimulated with IFN-γ.** (A and B) Cells were stimulated as indicated with IFN-γ. Following stimulation lysates were subjected to ChIP analyses and IP with control antibody or with antibody against histones H3 (A) or H4 (B). DNA was analyzed via Q-PCR with primers and probes spanning the CIITApIV IRF-E-GAS. IP values shown represent mean ± SEM of two independent experiments. Control IP values for (A) and (B) were 1.8±0.7 (C) IRF-1, STAT-1, pSTAT-1, EZH2 and CBP protein expression. MDA MB 435 variants were stimulated with IFN-γ, harvested and subjected to Western Blot analysis of indicated proteins. Results shown are representative of three independent experiments.
(TIF)

Figure S4 **Cell surface expression of MHC II in MDA MB 435, 435-Brain1, and 435-Lung2 cells treated with EZH2 siRNA.** Cells were plated, treated with control siRNA or with EZH2 specific siRNA, and were stimulated with IFN-γ for 36 hours. Following stimulation, cells were trypsinized, washed, and incubated with PE-labeled anti-human *HLA-DRA* antibody. Following antibody incubation, cells were fixed and PE cell surface staining was measured by FACS-Canto. Results shown are representative of three independent experiments.
(TIF)
