# Supplementary figures and images for: Dysregulated Recruitment of the Histone Methyltransferase EZH2 to the Class II Transactivator (CIITA) Promoter IV in Breast Cancer Cells
Source: PLoS One. 2012 Apr 26;7(4):e36013. doi: 10.1371/journal.pone.0036013 (PMC3338556; doi:10.1371/journal.pone.0036013)

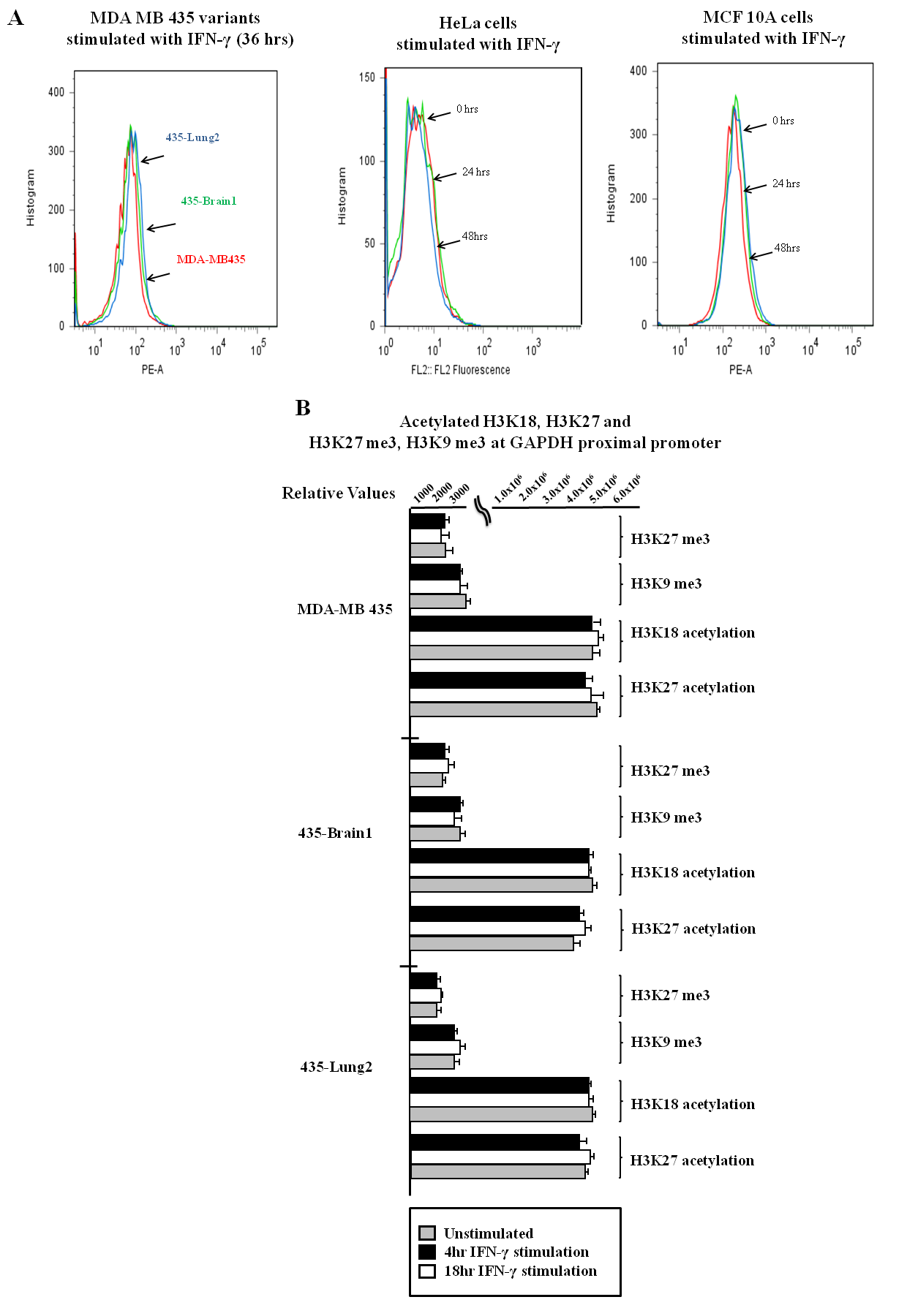

Supplement: Figure S1 — Isotype control staining of variants of MDA-MB 435, HeLa, and MCF 10A, and levels of H3K27me3, H3K9me3, H3K18ac, and H3K27ac at the GAPDH proximal promoter, are unaffected by cytokine stimulation in MDA MB 435, 435-Brain 1, and 435-Lung 2 cells. (A) MDA MB 435, 435-Brain 1, 435-Lung 2 cells, HeLa cells, and MCF 10A cells were stimulated with IFN-γ as indicated. Post stimulation cells were trypsinized, washed, and incubated with PE-labeled mouse control IgG. Following antibody incubation, cells were fixed and PE cell surface staining was measured by FACS-Canto. Results shown are representative of three independent experiments. (B) Cells were stimulated as indicated and were subjected to ChIP assay as above. Lysates were IP with isotype control antibodies or antibodies against indicated histone modification; associated DNA was isolated and analyzed via Q-PCR using primers spanning the GAPDH proximal promoter. Values shown represent mean ± SEM of two independent experiments. Average control IP values were 350±200 (TIF) [file pone.0036013.s001.tif]

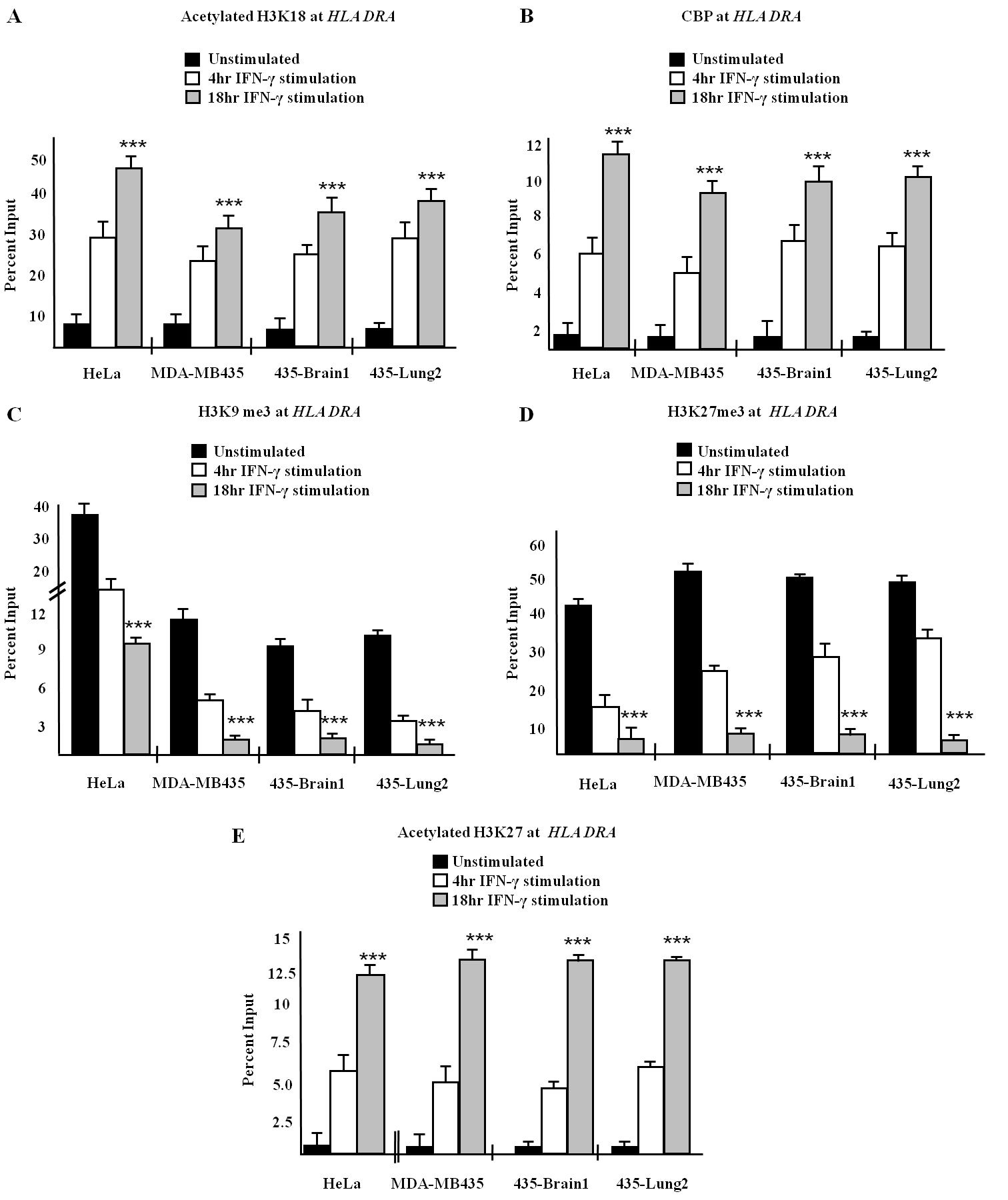

Supplement: Figure S2 — IFN-γ induced levels of H3K18ac, H3K27ac, CBP, H3K9me3, and H3K27me3 at the HLA-DRA promoter in HeLa cells and MDA MB 435 variants. (A–E) ChIP assays were carried out in HeLa cells, MDA MB 435 breast cancer cells, 435-Brain 1 cells, and 435-Lung2 cells stimulated as indicated with IFN-γ as described above. DNA was isolated and analyzed via Q-PCR using primers spanning the W-X-Y box of the HLA-DRA promoter. Values represent mean ± SEM of two independent experiments for HeLa and four independent experiments for MDA MB 435 variants. Average control IP values were 0.9±0.7 (TIF) [file pone.0036013.s002.tif]

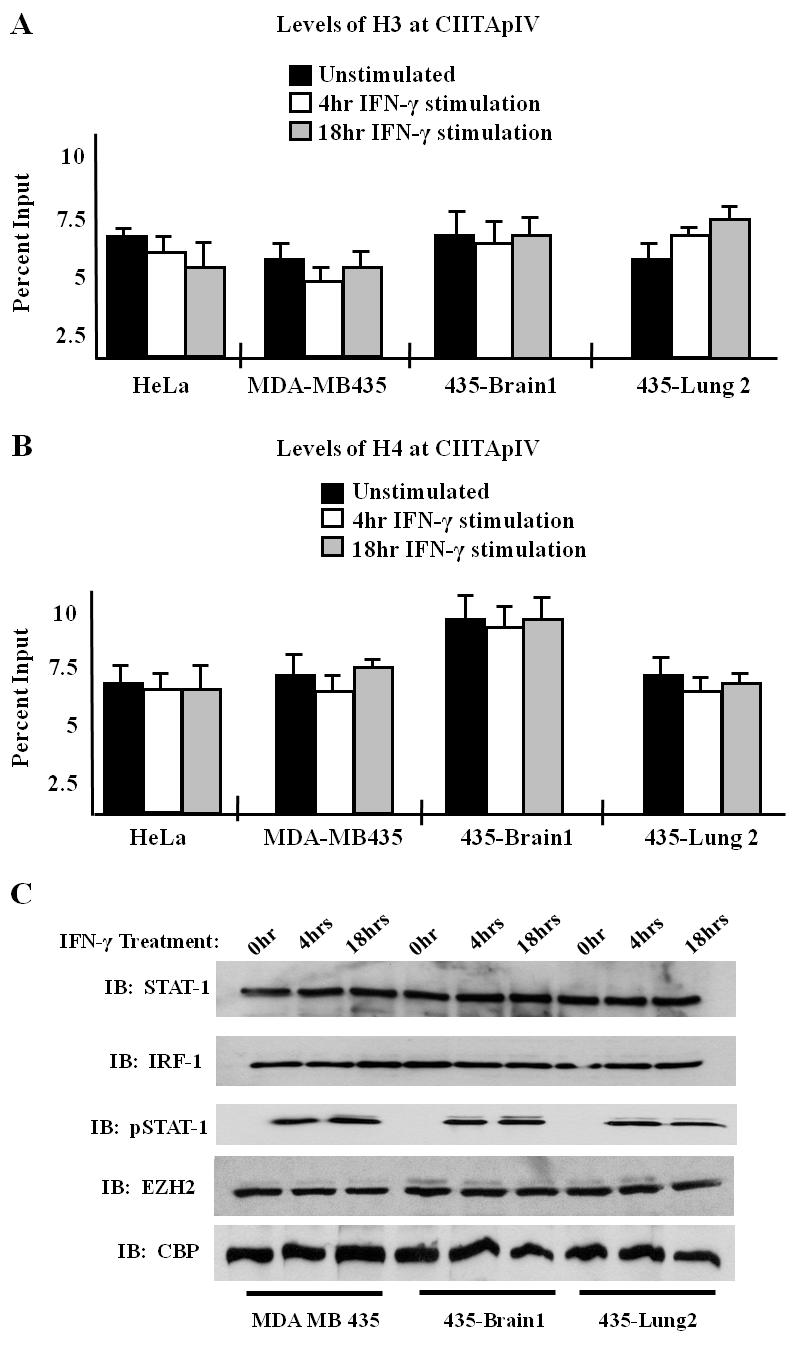

Supplement: Figure S3 — Levels of H3 and H4 remain unchanged in HeLa cells and in MDA MB 435 variants stimulated with IFN-γ. (A and B) Cells were stimulated as indicated with IFN-γ. Following stimulation lysates were subjected to ChIP analyses and IP with control antibody or with antibody against histones H3 (A) or H4 (B). DNA was analyzed via Q-PCR with primers and probes spanning the CIITApIV IRF-E-GAS. IP values shown represent mean ± SEM of two independent experiments. Control IP values for (A) and (B) were 1.8±0.7 (C) IRF-1, STAT-1, pSTAT-1, EZH2 and CBP protein expression. MDA MB 435 variants were stimulated with IFN-γ, harvested and subjected to Western Blot analysis of indicated proteins. Results shown are representative of three independent experiments. (TIF) [file pone.0036013.s003.tif]

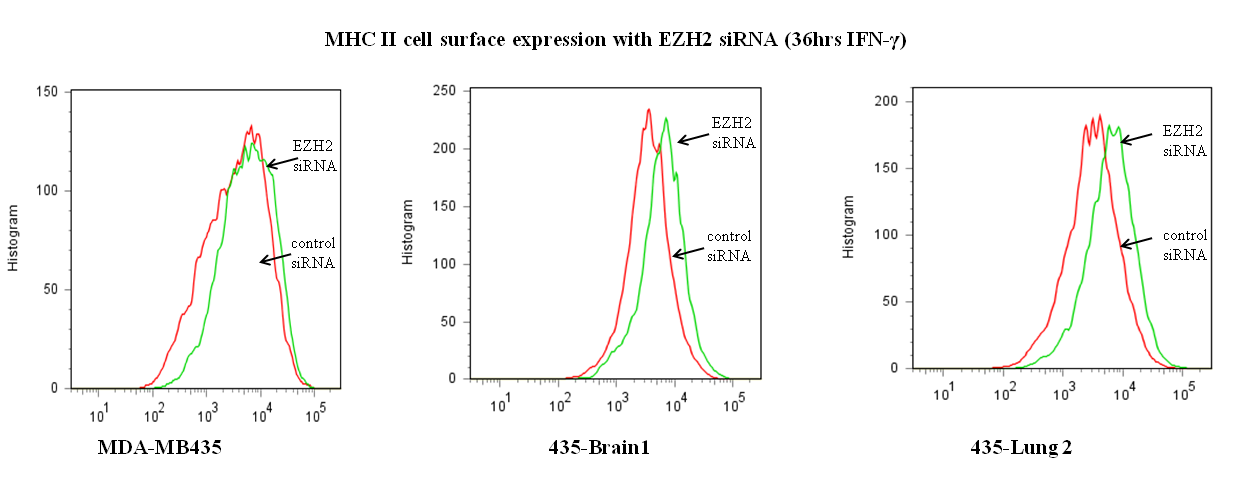

Supplement: Figure S4 — Cell surface expression of MHC II in MDA MB 435, 435-Brain1, and 435-Lung2 cells treated with EZH2 siRNA. Cells were plated, treated with control siRNA or with EZH2 specific siRNA, and were stimulated with IFN-γ for 36 hours. Following stimulation, cells were trypsinized, washed, and incubated with PE-labeled anti-human HLA-DRA antibody. Following antibody incubation, cells were fixed and PE cell surface staining was measured by FACS-Canto. Results shown are representative of three independent experiments. (TIF) [file pone.0036013.s004.tif]
